# Riboswitches

**DOI:** 10.1016/j.cub.2023.03.069

**Published:** 2023-05-08

**Authors:** Hubert Salvail, Ronald R. Breaker

**Affiliations:** 1 Department of Molecular, Cellular and Developmental Biology, Yale University, New Haven, CT 06511-8103, USA; 2 Department of Molecular Biophysics and Biochemistry, Yale University, New Haven, CT 06511-8103, USA; 3 Howard Hughes Medical Institute, Yale University, New Haven, CT 06511-8103, USA

**Keywords:** aptamer, expression platform, gene regulation, metabolite, noncoding RNA

Riboswitches are structured noncoding RNA domains that are typically found embedded in messenger RNAs where they sense specific target molecules or elemental ions and regulate gene expression. These RNAs thus serve as genetic switches that can activate or suppress gene expression in response to changing levels of their target ligand. To many observers, riboswitches might seem like rare oddities that are not as sophisticated as, or competitive with, the various protein factors that perform these same roles. However, as the number of experimentally validated riboswitch classes increases, and their true biochemical sophistication is recognized, it is becoming clearer that many species from all three domains of life entrust RNAs to make important chemical sensing and gene control decisions without the necessary participation of protein factors.

To date, more than 55 riboswitch classes have been experimentally validated, and the ligands they sense comprise a diverse list of biologically relevant elemental ions and fundamental metabolites that are commonly derived from RNA nucleotides or their precursors. This notable bias in the ligand specificities strongly suggests that the most common riboswitches of today’s organisms are descendant from ancient versions that first evolved in the ‘RNA World’, which is a proposed era of life when chemical transformations and molecular sensing were carried out predominantly by enzymes and receptors made of RNA.

Thus, by more closely examining the functions, structures, and mechanisms of riboswitches, it becomes possible to look back in time to understand more deeply how RNA World organisms survived, and even thrived, without the help of protein factors that have come to dominate these functions in modern organisms. This can reveal how a relatively simple nucleic acid polymer with only four common nucleotide components can form sophisticated molecular sensors and regulatory devices. In addition, each new riboswitch discovery exposes the collection of genes regulated in response to specific ligands, and this often reveals the functions of proteins whose activities had puzzled researchers sometimes for decades.

## The components of riboswitches and other RNA-based gene control devices.

Bacterial riboswitches usually reside in the 5′-untranslated region (UTR) of the mRNA whose production or expression they control. Typically, they are composed of two parts: (*i*) an aptamer that senses the target ligand, and (*ii*) an expression platform directly interfaces with components of the cell that affect gene expression. Natural aptamer domains are much like the synthetic aptamers first created by the laboratories of Larry Gold and Jack Szostak in the early 1990s. Although these early ‘test-tube evolution’ studies were instrumental in proving that RNAs could fold to form a great diversity of selective binding pockets for various biochemical ligands, it is now clear that natural evolution exploited these capabilities several billion years earlier.

**Figure 1.**
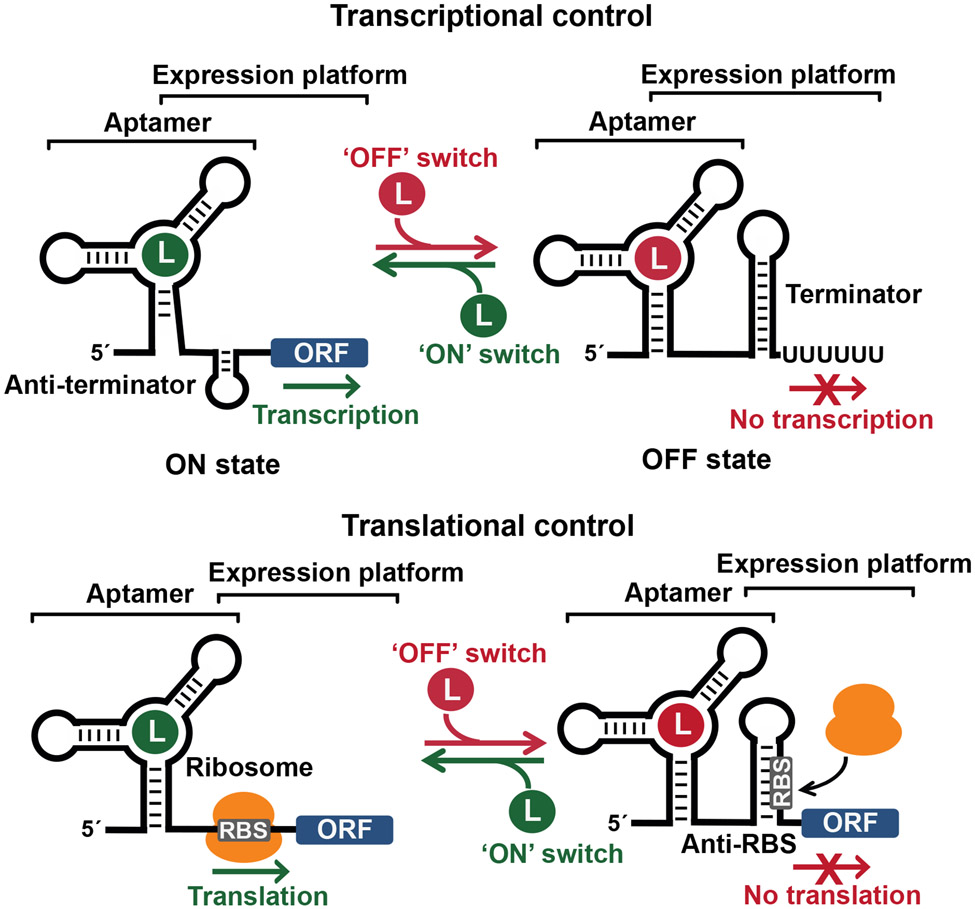
General riboswitch regulation mechanisms. Riboswitches rely on partially overlapping aptamer and expression platform domains to regulate gene expression at the transcriptional or translational levels. In the case of transcriptional control (top), ligand binding to the aptamer results in the formation of either an anti-terminator that promotes transcription elongation into the open-reading frame, thereby activating gene expression (‘ON’ switch), or of a terminator that halts transcription, and then prevents gene expression (‘OFF’ switch). For translational control (bottom), ligand binding to the aptamer renders the ribosome binding site either more accessible, thereby promoting translation initiation (‘ON’ switch), or less accessible, thus preventing translation (‘OFF’ switch). ORF, open reading frame; RBS, ribosome binding site.

Usually, each riboswitch aptamer partially overlaps an adjacent expression platform, wherein ligand binding to the aptamer alters the final structure of the expression platform in a manner that affects gene expression. Various types of expression platforms are exploited by riboswitches, but the two most common mechanisms involve regulation of premature transcription termination and control of ribosome binding to mRNAs (Figure 1). The first type of expression platform makes use of a simple RNA structure called an intrinsic transcription terminator. Terminators are hairpin structures formed by strong (mostly G-C) base-pairs, followed by a short, contiguous region of U nucleotides. These were found to be important components of gene regulation systems called attenuators in the 1970s by the laboratory of Charles Yanofsky. Attenuators modulate the expression of amino acid biosynthesis genes by exploiting an upstream open reading frame (uORF) containing multiple codons for a specific amino acid whose translation efficiency determines whether transcription proceeds to encompass the main ORF(s), or whether transcription halts within the run of U nucleotides of the intrinsic terminator element. For example, a deficit of tryptophan leads to a lower abundance of aminoacyl-tRNA^Trp^, which slows translation of ribosomes encountering repetitive Trp codons. Slow translation promotes an RNA folding pathway that prevents formation of the terminator stem, thereby promoting expression of genes whose protein products increase the production of tryptophan.

Despite some similarities in the components of riboswitches and attenuators, the two systems are distinct. A riboswitch aptamer directly and selectively binds the ligand, which then dictates the folding pathway taken by the terminator region or any other type of expression platform. In contrast, attenuators rely on an aminoacyl-tRNA synthetase to selectively sense the ligand, and then rely on ribosomes to detect the aminoacylated tRNA. Eventually, the speed of ribosome translation dictates expression platform folding. Thus, riboswitches that sense metabolites, other small molecules, or elemental ions are largely self-contained genetic devices that are the dominant participants in both their molecular sensing and gene control functions.

Another RNA-based regulatory system that monitors intracellular amino acid concentrations to regulate gene expression are T boxes that were first described by the laboratory of Tina Henkin in the early 1990s. T boxes are RNA elements that sense amino acids shortage through direct binding of nonaminoacylated tRNAs that in turn affects folding of a downstream expression platform. Here again, T box RNAs do not directly recognize the amino acids whose concentrations they are evaluating, but rather use Watson-Crick base-pairing and other RNA-RNA interactions to bind uncharged tRNAs that result when the target amino acid is low in concentration.

## Basic and complex gene control functions of riboswitches.

Most known riboswitches function as genetic “OFF” switches, wherein ligand binding suppresses gene expression. This type of feed-back control is common because there is often a need for cells to turn off expression of genes for metabolite biosynthesis or import when a desirable biomolecule is abundant. For example, AdoCbl riboswitches (sense coenzyme B_12_) of some bacterial species are known to turn off the expression of the large operon that codes for most of the 25 genes required for biosynthesis of its target enzyme cofactor. Thus, when coenzyme B_12_ concentrations are adequate, the cell saves the resources that it otherwise would commit to making these biosynthesis proteins when the coenzyme concentration is low.

There are also many examples of riboswitches that operate as genetic “ON” switches. For example, riboswitches for fluoride and others for guanidine activate the expression of genes coding for exporters of these toxic chemicals to mitigate their effects. Another example of an unusual genetic ON switch is represented by glycine riboswitches. These RNAs carry two aptamers that work cooperatively to activate the *gcvT* operon, which codes for the proteins forming the glycine cleavage system. When cells accumulate excess glycine, the riboswitch triggers the cell to degrade this amino acid and route its carbon through the citric acid cycle.

Glycine riboswitches were the first examples wherein components of riboswitches or even multiple riboswitches were found reside in tandem to create more sophisticated genetic switches. For example, instead of the more common ‘one aptamer/one expression platform’ arrangement, complete riboswitches are frequently observed to reside in tandem with other riboswitches to control the expression of an adjacent gene. Tandem aptamers that bind the same ligand yield steeper dose-response curves to changing metabolite concentrations, whereas those having different ligand specificities yield natural Boolean logic gates that evaluate two chemical inputs when making gene expression decisions. Even more complex arrangements are known including rare examples of allosteric ribozyme-riboswitches, wherein ligand binding to an aptamer regulates RNA processing by an adjacent self-splicing ribozyme.

## How abundant are riboswitches?

**Figure 2.**
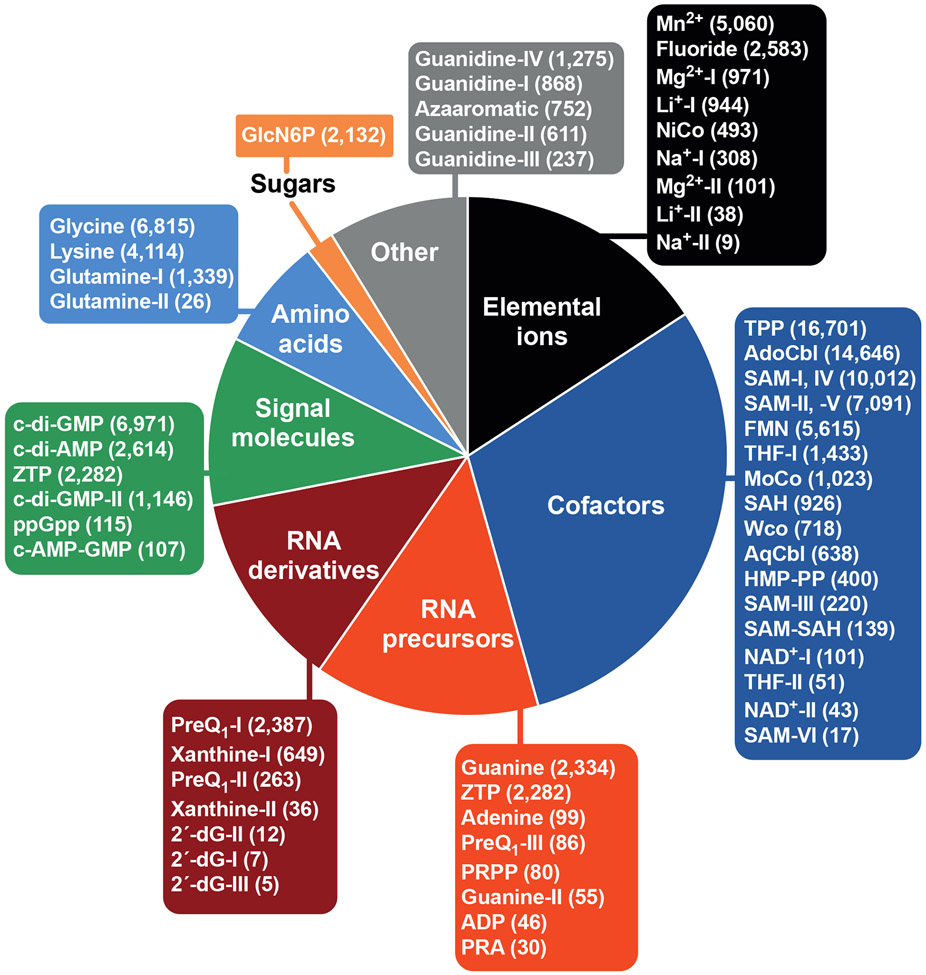
List of experimentally validated riboswitches. The plotted data correspond to the abundances of riboswitch classes according to the biochemical category of their cognate ligand. Values in parentheses indicate the number of representatives for each riboswitch class. 2′-dG, 2′-deoxyguanosine; ADP, adenosine 5′-diphosphate; AdoCbl, adenosylcobalamin; AqCbl, aquocobalamin; c-di-AMP, cyclic diadenosine monophosphate; c-di-GMP, cyclic diguanosine monophosphate; c-di-AMP-GMP, cyclic adenosine monophosphate-guanosine monophosphate; FMN, flavin mononucleotide; GlcN6P, glucosamine-6-phosphate; HMP-PP, 4-amino-2-methyl-5-hydroxymethylpyrimidine pyrophosphate; MoCo, molybdenum cofactor; NAD^+^, nicotinamide adenine dinucleotide; ppGpp, guanosine tetraphosphate; PRA, phosphoribosylamine; preQ_1_, prequeuosine-1; PRPP, phosphoribosyl pyrophosphate; SAH, *S*-adenosylhomocysteine; SAM, *S*-adenosylmethionine; THF, tetrahydrofolate; TPP, thiamin pyrophosphate; WCo, tungsten cofactor; ZTP, 5-amino 4-imidazole carboxamide riboside 5′-triphosphate.

Since the first experimental validation studies on riboswitches were published in 2002, more than 55 riboswitch classes have been reported. Each riboswitch is classified according to the ligand it senses and/or the distinct structures it uses to form the aptamer domain. Some ligands, such as *S*-adenosylmethionine (SAM) and guanidine are sensed by several riboswitch classes that use profoundly different aptamer structures to sense the same target molecule. Also observed are riboswitches that carry sometimes very subtle mutations in the aptamer domain that alter ligand specificity. This is strikingly represented by riboswitches for guanidine, the signaling molecule ppGpp, the RNA biosynthetic intermediate PRA, and the RNA nucleotide ADP, as these four ligands are selectively recognized by different variants that share a common aptamer architecture. Thus, by exploiting distinct or similar aptamer architectures, the known riboswitch classes bind numerous fundamental metabolites, signaling molecules, toxic chemicals, and elemental ions (Figure 2).

Furthermore, there many ‘orphan’ riboswitch candidates that exhibit various features that are characteristic of known riboswitches, but whose ligands have yet to be established. Researchers attempting to validate these candidates typically encounter various challenges that have frustrated efforts to identify their ligands. The genes associated with riboswitch candidates often can provide clues regarding the identity of the riboswitch ligand, but not if the function of its protein product has been misannotated. Similarly, genetic screens often can reveal functional links between additional genes and the riboswitch ligand, but this information can be unhelpful if the details of these genes and their roles in metabolism are not well understood. Successful experimental validation of some orphans might require improvements in the computational tools used for gene annotation or the use of additional unbiased experimental approaches for matching newly identified riboswitches with their ligand. Although it can be difficult to elucidate the function of additional riboswitch classes, there is much to gain by increasing knowledge regarding novel areas of biology, as described in more detail later.

It seems certain that the current list of validated and orphan riboswitch classes represents only a tiny fraction of the total number present across extant bacterial species. This prediction is based on the observation that the collection of known riboswitch classes largely follows a power law distribution based on their abundances is organisms whose genomes have been sequenced and analyzed. This projection indicates that many thousands of riboswitch classes remain hidden in bacteria, although many of these classes might be sparsely distributed among only a few species. If true, then as novel riboswitch finds are made over time, they are likely to become progressively rarer. This means that the process of discovering additional classes will become progressively more difficult, which might require novel search strategies and methods to sustain.

**Figure 3.**
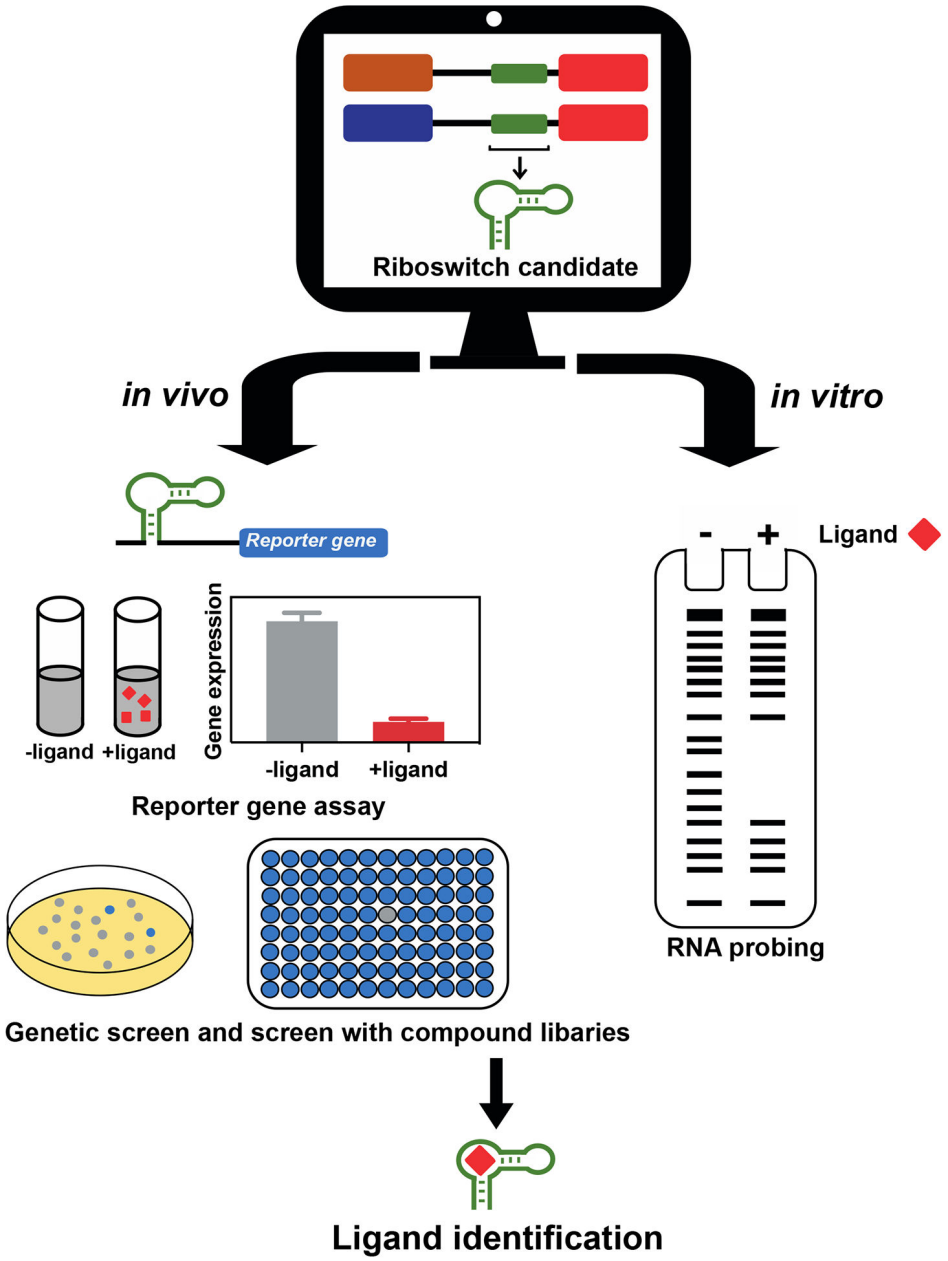
General experimental procedure for riboswitch ligand discovery. Riboswitch candidates are typically identified through comparative genomics analyzes. Putative ligands inferred from candidates’ gene associations are then evaluated for their ability to alter the activity of a riboswitch gene reporter construct and/or for direct binding to a representative RNA of the candidate by RNA probing. Genetic screens or screens with compounds libraries are performed when predicting the ligand of a riboswitch candidate is challenging or when validation attempts with ligand candidates fail.

Currently, the process of discovering new riboswitch candidates typically begins by using comparative sequence analysis algorithms to identify intergenic regions that exhibit sequence and structural conservation among various species (Figure 3). Putative ligand compounds, inferred from the biological function of the genes associated with the selected candidates, are then tested for direct binding to RNA representatives of a given motif using RNA structure probing and for their ability to modulate gene expression when fused to a reporter gene. In cases where the ligand cannot be easily inferred from gene association or validation efforts with a list of candidate ligands fail, biochemical testing with compound libraries or cellular extracts, or genetic screens, are performed with the goal of identifying the ligand.

## Are riboswitches descendants of the RNA World?

There are several lines of evidence supporting the hypothesis that some riboswitches might be modern descendants of the ‘RNA World’, a proposed era in evolution during which RNA both served as a medium for genetic information storage and as a medium for forming functional structures such as enzymes and receptors. These RNA functions would have been necessary for sophisticated biological systems to have been based on RNA, long before DNA and proteins emerged to accomplish these two broad functions. This hypothesis is partially supported by the observation that many riboswitch classes recognize fundamental RNA-derived molecules such as enzyme cofactors, RNA nucleotides and their precursors or derivatives, and RNA-based signal molecules (e.g., ppGpp, c-di-AMP, c-di-GMP) (Figure 2). These compounds could be relics from the RNA World, in which they would have played important biological roles in ancient forms of life as they currently do in extant organisms.

Even if many riboswitch classes did not emerge in the RNA World, their modern architectures and functions nevertheless are reflective of the capabilities that could have been exploited by primitive forms of life. The known riboswitch classes recognize a large variety of ligands, which highlights the incredible structural ability of RNA to form binding pockets that sense a great diversity of biologically relevant chemicals. Just as seen in modern cells, RNA World organisms could have used this capacity for molecular recognition to monitor and regulate fundamental biochemical processes. Also, as noted above, numerous riboswitches selectively bind RNA-derived cofactors that many protein enzymes use to catalyze essential biochemical reactions. Perhaps coenzyme biding sites like those observed in modern riboswitches were used by RNA World ribozymes to catalyze chemical transformations long before protein enzymes competed for these same functions.

## Why study riboswitches?

After more than two decades of riboswitch research, dozens of riboswitch classes have been uncovered and examined. Thus, one may question the value of finding additional novel riboswitch classes and exploring their mechanistic and structural details. However, recent discoveries have revealed riboswitches that sense surprising ligands to reveal biological processes that were not known or at least not well understood. Notably, the validation of new candidates also allowed to identify the function of genes that were misannotated, or for which the biological function had remained unknown. For example, the discovery of fluoride riboswitches (formerly known as the *crcB* RNA motif) led to the functional reannotation of genes associated with the motif that had no obvious link with fluoride toxicity resistance. Fluoride riboswitches commonly regulate genes annotated as *eriC*. Many *eriC* genes code for chloride transporter proteins, but the common association of fluoride riboswitches revealed that some EriC proteins have acquired mutations that altered the specificity to favor fluoride transport. Similarly, the discovery of guanidine riboswitches associated with genes whose protein products were previously annotated as urea carboxylase enzymes or multidrug efflux channels indicated a problem with these assigned functions. Indeed, these proteins were soon demonstrated to function as guanidine carboxylase enzymes and guanidine transporters, respectively.

The discovery of new riboswitch classes also often reveals novel mechanisms by which bacteria integrate environmental and metabolic changes to influence gene expression, thereby adapting to these variations. These advances have revealed the common mechanisms by which riboswitches control gene expression (Figure 1), but also have revealed the existence of riboswitches that have more complex functions, including operating in tandem as Boolean logic devices. By ‘reverse engineering’ these natural systems, new synthetic RNA sensors and switches could be made better for promising therapeutic and biotechnological applications.

One envisioned application of designer riboswitches is to use them as components of gene therapy constructs to modulate the expression of transgenes when delivered in humans. Such riboswitches could be made responsive to natural metabolites or to synthetic compounds that could be administrated as drug-like modulators of the therapeutic transgene. Although numerous RNA switches with aptamers engineered to be selective for novel ligands have been developed, it remains challenging to create synthetic riboswitches that exhibit performance characteristics that are meet the challenges for utility in humans. Perhaps, the lessons learned from studying natural riboswitches will aid in the process of engineering riboswitches with the desired characteristics.

Riboswitch research also has paved the way for researchers to consider these RNA devices as druggable targets for antibacterial agent development. Riboswitches that naturally bind small molecule ligands should present drug developers with RNA structures that can be bound by ligand analogs or other small molecules that trick the riboswitch into triggering changes in gene expression that normally would only be achieved when the natural ligand binds. For example, a genetic OFF switch could be exploited by binding an analog that turns off the expression of a gene whose protein product would otherwise boost the concentration of an essential metabolite. In this manner, a riboswitch-targeting drug could cause cell distress or death by starving the cell for the natural metabolite.

Novel compounds that target riboswitches and prevent bacterial cell growth have been identified, and several have been tested in animal models where they suppress bacterial infections. However, there are limitations to the development and use of riboswitch-targeting antibacterial agents. Only a few riboswitch classes are widespread among pathogenic bacterial species, which hinders the development of broad-spectrum antibiotics. Furthermore, it is likely that some bacterial species would rapidly overcome the effects of the antibiotic simply by acquiring mutations in the riboswitch binding site, thereby avoiding the suppression of essential genes. These challenges, in addition to the high costs inherent to the development of new classes of antimicrobial compounds, has hindered the exploitation of bacterial riboswitches as drug targets.

## The future of riboswitch research

The last two decades have been a productive era for riboswitch discovery and analysis. Through these efforts, it is apparent that riboswitches are extensively used by many bacterial species to regulate numerous genes for major metabolic pathways and physiological processes. It is no longer surprising to learn that bacteria entrust another riboswitch class to sense some important ligand and regulate genes relevant to a key biological process. For many bacterial species, riboswitches and protein factors share the ligand sensing and gene regulation tasks as near equal contributors. Future research efforts are likely to uncover many additional classes that will fill some unexpected gaps on the list of known riboswitch classes. Notably, riboswitches that selectively sense compounds relevant to phosphate homeostasis, sugar metabolism, lipid metabolism, and biosynthesis of essential cofactors like coenzyme A, pyridoxal phosphate, and biotin have yet to be discovered. Although some of these riboswitch ‘blind spots’ could be reflective of the limited molecular sensing capabilities of RNA, it is likely that riboswitch classes for most of these ligands simply remain to be found.

Another topic that deserves special attention is related to the use of riboswitches by eukaryotes. Most riboswitches have so far been identified exclusively in bacteria, which highlights the following question: do eukaryotes make extensive use of riboswitches for metabolites or elemental ions? In bacteria, riboswitches for the enzyme cofactor thiamin pyrophosphate (TPP) riboswitch are strikingly abundant. Perhaps not surprisingly, TPP riboswitches are also abundant in fungi and plants, where they often regulate gene expression by controlling alternative splicing and other RNA processing events. Likewise, it seems certain that other riboswitch classes will be found in eukaryotic species where they will regulate gene expression using similar mechanisms as that observed for TPP-sensing RNAs.

However, bioinformatic searches in eukaryotic genomes for homologs of other bacterial riboswitches have so far failed to uncover candidates that are worth investigating. Also, occasional claims of metabolite-responsive riboswitches in eukaryotes based on biochemical or genetic results have not been confirmed, which has dampened enthusiasm for the prospects of finding abundant examples in these genomes. The challenge of finding and validating riboswitches in eukaryotes is due in part to the large sizes of their genomes and the vast regions of noncoding sequences compared to bacteria. Whereas bacterial riboswitches almost always reside in the 5′-UTR of an mRNA, the known eukaryotic representatives are in 5′-UTRs, introns, and 3′-UTRs, thus expanding the search space greatly.

Successful large-scale riboswitch discovery in any of the domains of life will likely depend on refining the current experimental and computational validation approaches. The approaches that have proven to be effective for uncovering over 55 riboswitch classes cannot maintain the previous pace of discovery in part because the remaining riboswitch classes in bacteria are likely to be distributed among only a few bacterial species. In eukaryotes, the vast search space and likely novel RNA structures present will also frustrate those who are using conventional search approaches. However, efforts to improve the search methods will undoubtedly be rewarded by the discovery of novel riboswitches that will teach us new lessons about ligand sensing, gene regulation, and biological processes that can best be learned by investigating these RNA devices.
